# Variability of CSF Alzheimer’s Disease Biomarkers: Implications for Clinical Practice

**DOI:** 10.1371/journal.pone.0100784

**Published:** 2014-06-24

**Authors:** Stephanie J. B. Vos, Pieter Jelle Visser, Frans Verhey, Pauline Aalten, Dirk Knol, Inez Ramakers, Philip Scheltens, Marcel G. M. Olde. Rikkert, Marcel M. Verbeek, Charlotte E. Teunissen

**Affiliations:** 1 Department of Psychiatry and Neuropsychology, Maastricht University, School for Mental Health and Neuroscience, Alzheimer Center Limburg, Maastricht, the Netherlands; 2 Alzheimer center & Department of Neurology, Neuroscience Campus Amsterdam, VU University Medical Center, Amsterdam, The Netherlands; 3 Department of Epidemiology and Biostatistics, VU University Medical Center, Amsterdam, the Netherlands; 4 Department of Geriatric Medicine, Radboud University Nijmegen Medical Centre, Nijmegen, The Netherlands; 5 Donders Institute for Brain, Cognition and Behaviour, Radboud University Nijmegen and Radboud Alzheimer Centre, Nijmegen, The Netherlands; 6 Department of Neurology and Department of Laboratory Medicine, Radboud University Nijmegen Medical Centre, Nijmegen, The Netherlands; 7 Department of Clinical Chemistry, Neurological Laboratory, VU University Medical Center, Amsterdam, The Netherlands; University of Verona, Italy

## Abstract

**Background:**

Cerebrospinal fluid (CSF) biomarkers are increasingly being used for diagnosis of Alzheimer’s disease (AD).

**Objective:**

We investigated the influence of CSF intralaboratory and interlaboratory variability on diagnostic CSF-based AD classification of subjects and identified causes of this variation.

**Methods:**

We measured CSF amyloid-β (Aβ) 1-42, total tau (t-tau), and phosphorylated tau (p-tau) by INNOTEST enzyme-linked-immunosorbent assays (ELISA) in a memory clinic population (n = 126). Samples were measured twice in a single or two laboratories that served as reference labs for CSF analyses in the Netherlands. Predefined cut-offs were used to classify CSF biomarkers as normal or abnormal/AD pattern.

**Results:**

CSF intralaboratory variability was higher for Aβ1-42 than for t-tau and p-tau. Reanalysis led to a change in biomarker classification (normal vs. abnormal) of 26% of the subjects based on Aβ1-42, 10% based on t-tau, and 29% based on p-tau. The changes in absolute biomarker concentrations were paralleled by a similar change in levels of internal control samples between different assay lots. CSF interlaboratory variability was higher for p-tau than for Aβ1-42 and t-tau, and reanalysis led to a change in biomarker classification of 12% of the subjects based on Aβ1-42, 1% based on t-tau, and 22% based on p-tau.

**Conclusions:**

Intralaboratory and interlaboratory CSF variability frequently led to change in diagnostic CSF-based AD classification for Aβ1-42 and p-tau. Lot-to-lot variation was a major cause of intralaboratory variability. This will have implications for the use of these biomarkers in clinical practice.

## Introduction

Amyloid-β (Aβ) 1-42, total tau (t-tau), and phosphorylated tau (p-tau) proteins in cerebrospinal fluid (CSF) are well-established biomarkers for Alzheimer’s disease (AD) [1]–[3], and are increasingly being used for diagnosis in clinical practice. Previous studies reported considerable intra- or interlaboratory variability of CSF analyses [4]–[7], which may influence the diagnostic classification. In this study, we performed a large-scale CSF multicenter study and investigated the exact influence of intra- and interlaboratory variability on CSF-based AD classification of subjects.

We hypothesized that the change of diagnosis would be largest for classification of subjects based on CSF Aβ1-42, as previous studies showed larger variability for CSF Aβ1-42 than for t-tau and p-tau [5]–[7]. It was also hypothesized that change of diagnosis would be lower for analyses performed in the same laboratory than for analyses performed in different laboratories because CSF intralaboratory variability has been reported to be lower than interlaboratory variability (2.3–25% vs. 13–38%) [5]–[7].

We investigated intralaboratory and interlaboratory variability of CSF Aβ1-42, t-tau, and p-tau analyses by INNOTEST enzyme-linked-immunosorbent assays (ELISA). Samples were measured twice in one or two laboratories that served as reference labs for AD CSF analyses in the Netherlands. We classified subjects based on validated cut-offs and examined how often CSF-based AD diagnosis changed after the second analysis.

## Materials and Methods

### CSF Samples

CSF samples were collected from subjects included in the Leiden Alzheimer Research Netherlands (LeARN) study [8]. LeARN is a Dutch multicenter study performed in a memory-clinic setting that included subjects between October 2009 and May 2011 that had been newly referred for the assessment of cognitive complaints. Inclusion criteria were baseline diagnosis of subjective cognitive impairment (SCI), mild cognitive impairment (MCI) or dementia, Mini-Mental State Examination (MMSE)≥20, clinical dementia rating scale (CDR) of maximal 1. Exclusion criteria were somatic, psychiatric or neurological disorders that could have caused the cognitive impairment.

In 3 of the 4 participating centers (Amsterdam, Maastricht, and Nijmegen), 126 CSF samples were analyzed in twofold, the first time as part of clinical routine and secondly for the LeARN study (Figure S1). For routine practice, samples were analyzed in Amsterdam and Nijmegen, which serve as national CSF AD biomarker centers. For the LeARN study, all samples were analyzed in a single batch in Amsterdam. The samples collected in Amsterdam (n = 50) were measured twice in the same lab and were used to assess intralaboratory variability. Samples collected in Nijmegen (n = 32) and Maastricht (n = 44) were measured twice in different laboratories, i.e. in Nijmegen for clinical routine and in Amsterdam for the LeARN study, and were used to study interlaboratory variability (total n = 76). Table S1 provides baseline patient demographics. The medical research ethics committee in Maastricht and the institutional review boards of Maastricht University Medical Center, VU Univeristy Medical Center Amsterdam, Radboud University Nijmegen Medical Center, and Leiden University Medical Center approved the study. All subjects provided written informed consent.

### CSF Procedures and Analyses

CSF was obtained by lumbar puncture between the L3/L4 or L4/L5 intervertebral space, and collected and aliquoted into polypropylene tubes. Samples for clinical routine of Maastricht were transported the same day on room temperature or stored at −20°C and transported on dry ice within one week to the Nijmegen laboratory for analysis. Samples for clinical routine analysis were stored at −20°C for up to 4 weeks (Amsterdam cohort) or at −80°C for up to 2 weeks (Maastricht/Nijmegen cohort) before analysis. Research samples (i.e. samples of the LeARN study) were stored at −80°C [9], at each center and samples of Maastricht and Nijmegen were transported on dry ice to Amsterdam for analysis after up to 2.5 years. Both laboratories used the commercially available INNOTEST enzyme-linked immunosorbent assays (ELISAs; Innogenetics, Ghent, Belgium) to quantify CSF Aβ1-42, CSF t-tau, and CSF p-tau, all performed by experienced laboratory technicians. For analysis of the research samples, the same lot number was used for all analyses, while for clinical routine analyses different lots were used in Nijmegen as well as in Amsterdam. Due to insufficient fluid material, CSF p-tau values were only available for 49 samples for intralaboratory analyses.

We also analyzed internal control samples from the Amsterdam lab to investigate the influence of lot-to-lot variation on measured CSF concentrations. One control sample had an AD typical profile and the other a normal CSF profile. The internal controls were aliquots for single use obtained by pooling surplus CSF. These aliquots were stored at −80°C and all internal controls used in the current study were from the same batch of pools.

To study differences in biomarker classification as normal versus abnormal between CSF measurements, we dichotomized the CSF variables according to routinely used validated cut-offs of each lab. In Amsterdam, cut-offs were determined that could differentiate subjects with SCI from subjects with AD-type dementia with 85% sensitivity: CSF Aβ1-42≤550 pg/ml, t-tau>375 pg/ml, and p-tau>52 pg/ml [10]. In Nijmegen, cut-offs were determined that could differentiate cognitively normal controls from subjects with AD-type dementia with a specificity of 95%: CSF Aβ1-42<500 pg/ml, t-tau>350 pg/ml, and p-tau>85 pg/ml [11]. Given that a different approach was used to define cut-offs in Amsterdam and Nijmegen, we performed interlaboratory analyses with the same cut-offs (Amsterdam cut-offs) as well as with lab-specific cut-offs (Amsterdam and Nijmegen cut-offs).

### Statistical Analyses

Statistical analyses were done with SPSS version 19.0 (Chicago, IL, USA) and GraphPad Prism 5, with significance set at p<0.05. Intralaboratory and interlaboratory coefficients of variation (CV) were calculated as the standard deviation (SD) divided by the mean of the measurements of each sample for each biomarker. Subsequently, a mean CV was calculated. We performed paired t-tests to investigate the intralaboratory and interlaboratory variability between CSF analyses of CSF Aβ1-42, t-tau, and p-tau. In addition, we calculated Pearson correlations *r* and Intraclass Correlation Coefficients (ICC). An ICC score ranges from 0 to 1, representing virtually no (0.00–0.10), a slight (0.11–0.40), fair (0.41–0.60), moderate (0.61–0.80), or substantial (0.81–1.00) level of agreement between the analyses [12]. Confidence intervals were calculated for both the Pearson correlations and ICC scores. We made Bland-Altman plots to visualize the agreement between the CSF analyses [13], and calculated the percentage of subjects with change of AD marker classification after the second analysis using routine CSF cut-offs of each laboratory. Interlaboratory lot-to-lot variation was examined using Deming regression [14].

## Results

### Intralaboratory Variability

**Figure 1:**
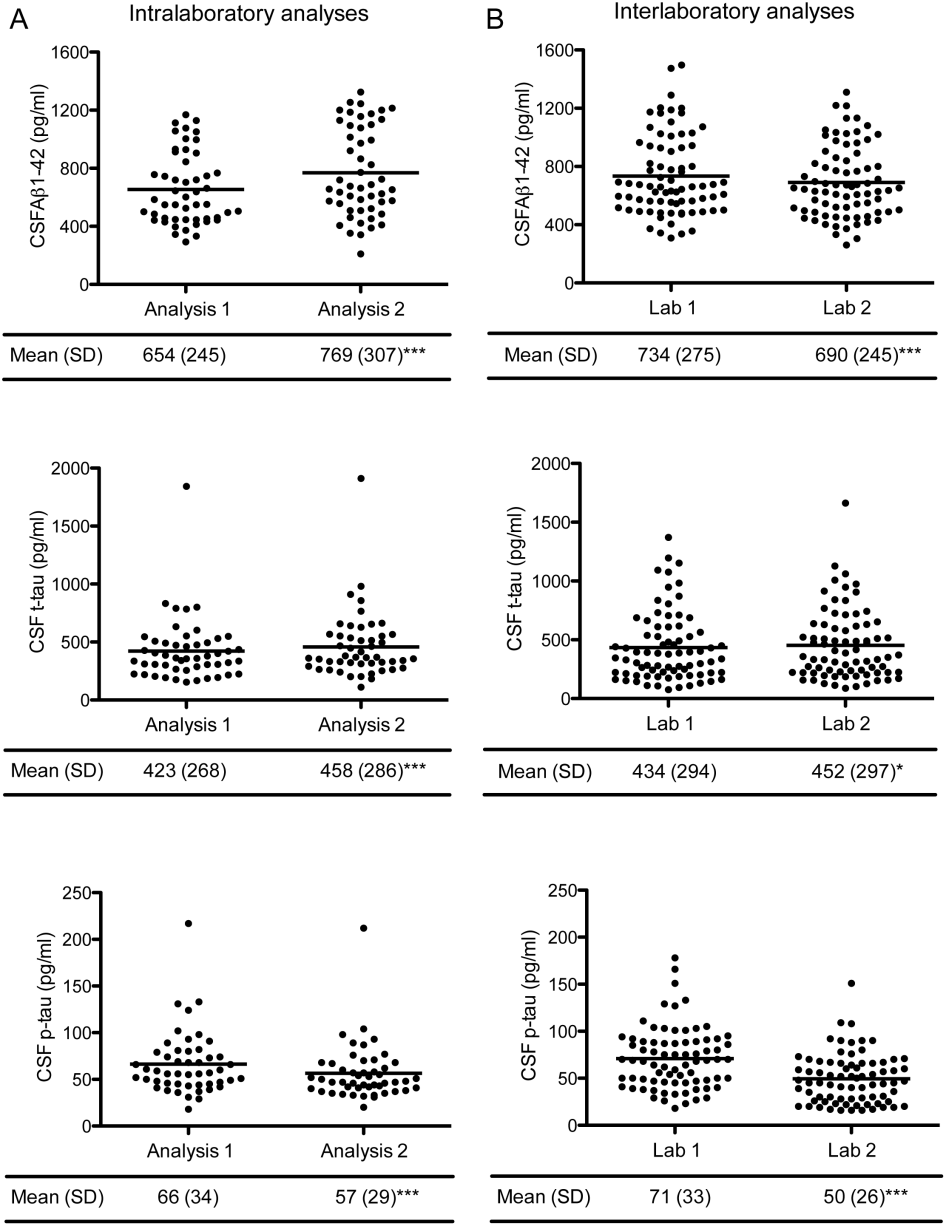
CSF levels by analysis and marker. Results are frequencies and mean (SD) for each CSF marker on the left (A) for CSF intralaboratory analyses and on the right (B) for CSF interlaboratory analyses. The solid line represents the mean CSF levels. Analysis 1 is routine practice and analysis 2 is performed as part of the LeARN study. CSF = cerebrospinal fluid, Aβ = amyloid beta, t-tau = total tau, p-tau = phosphorylated tau. **P<0.001, *p<0.05 compared to CSF analysis 1 or lab 1.

**Figure 2:**
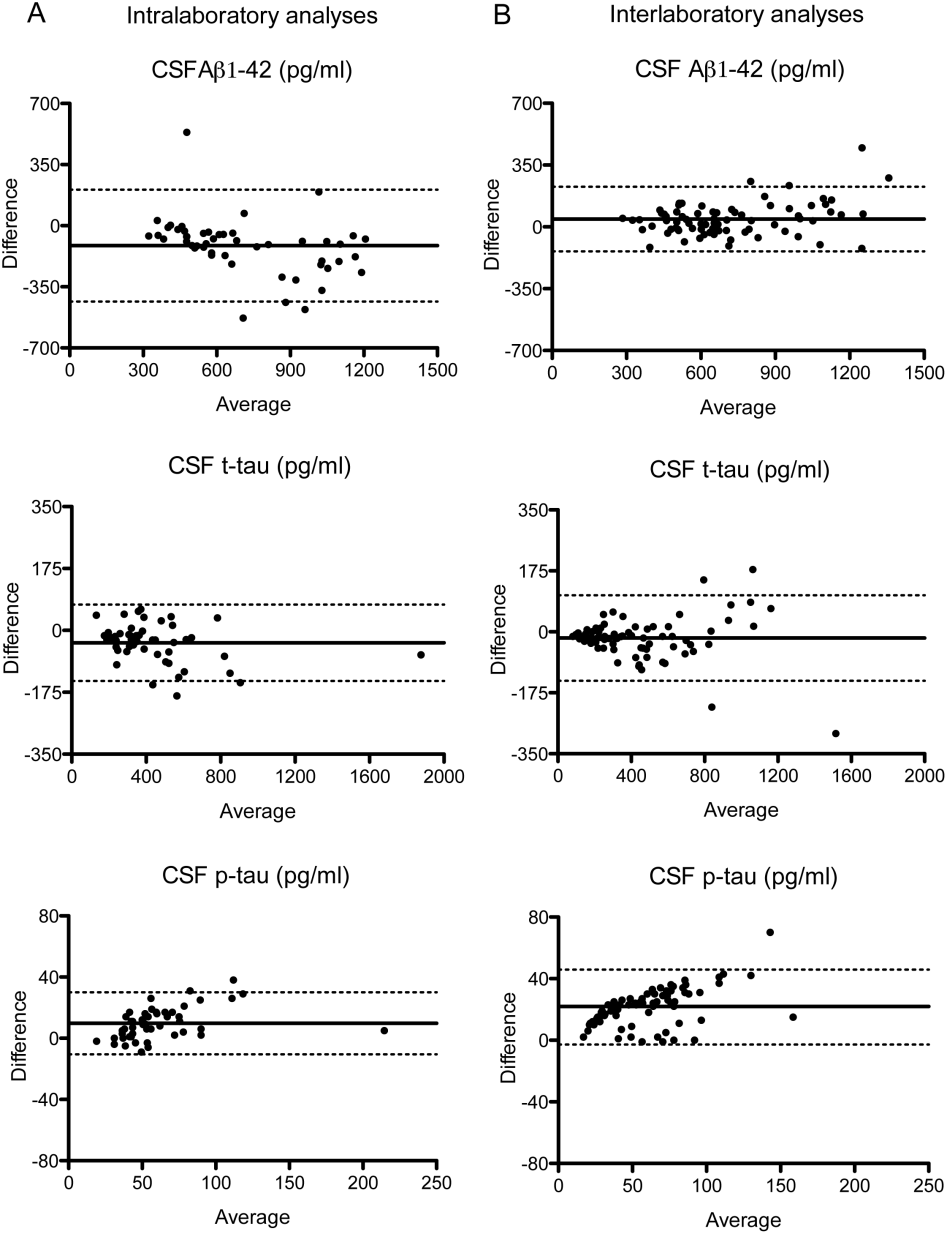
Bland-Altman plots of variability between CSF analyses. The average of CSF analysis 1 and 2 is plotted against the difference between both analyses, on the left (A) for CSF intralaboratory analyses and on the right (B) for CSF interlaboratory analyses. The solid line represents the mean and the dotted lines the upper and lower 1.95 SD. CSF = cerebrospinal fluid, Aβ = amyloid beta, t-tau = total tau, p-tau = phosphorylated tau.

**Figure 3:**
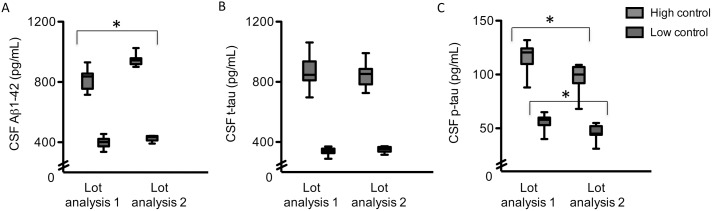
Internal control data of the Amsterdam laboratory. Results are changes in high and low CSF biomarker levels for intralaboratory reanalysis in two lots (routine lot and LeARN lot) as part of internal control of data at the Amsterdam laboratory. A) Aβ1-42: Lot 1 used from February 2010 to February 2011 (n = 18) and lot 2 used from March to October 2012 (n = 17). B) T-tau: Lot 1 used from February 2010 to April 2011 (n = 24) and lot 2 used from March to June 2012 (n = 11). C) P-tau: Lot 1 used from February 2010 to April 2011 (n = 23) and lot 2 used from March to October 2012 (n = 11). CSF = cerebrospinal fluid, Aβ = amyloid beta, t-tau = total tau, p-tau = phosphorylated tau. *P<0.001 for differences between lot of analysis 1 (routine lot) and lot of analysis 2 (LeARN lot).

**Figure 4:**
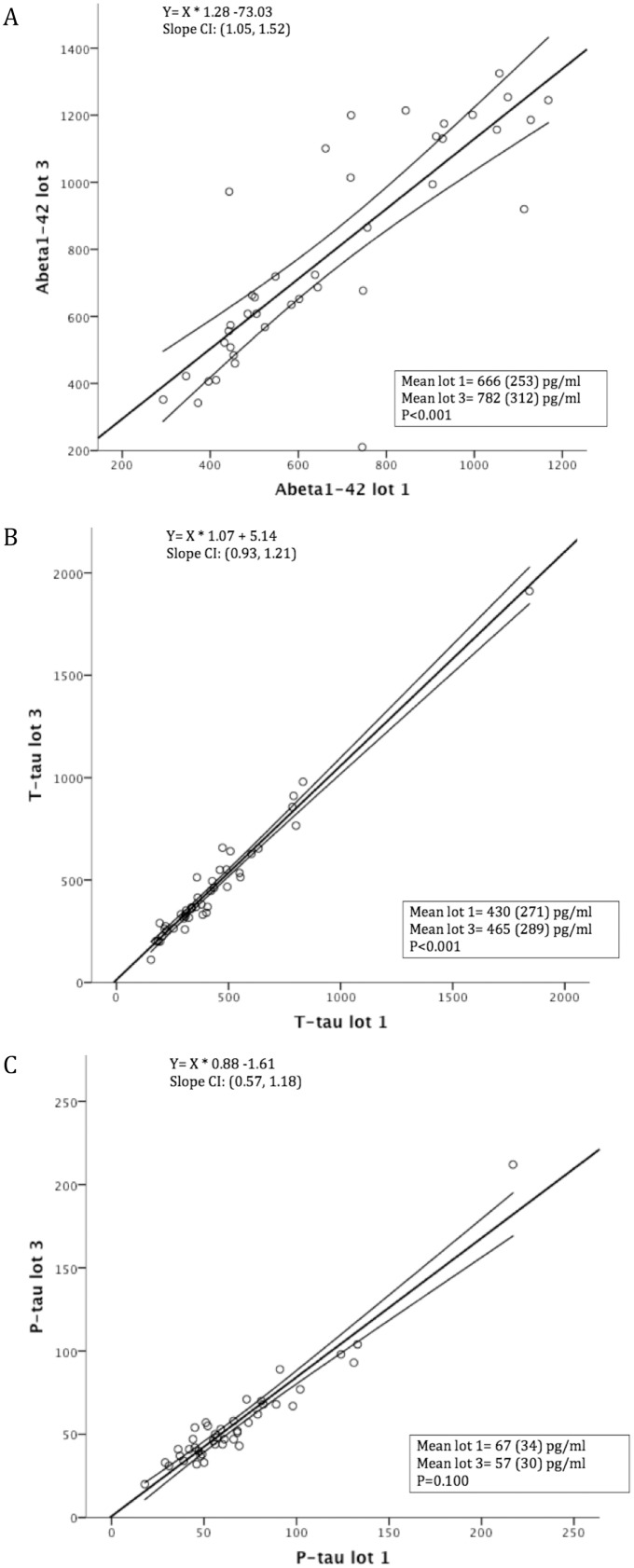
Intralaboratory lot-to-lot variation. Results in this graph are based on deming regression and show the CSF levels of lot 1 and lot 3 for Aβ1-42 (A), t-tau (B), and p-tau (C). The slope is different for Aβ1-42 levels between lot 1 and lot 3. The mean difference in CSF levels between lot 1 and 3 was significantly different for Aβ1-42 and t-tau. Lot 1 = clinical routine lot, lot 3 = LeARN lot, Aβ = amyloid beta, t-tau = total tau, p-tau = phosphorylated tau.

CSF Aβ1-42 and t-tau levels were higher and p-tau levels were lower after reanalysis in the same laboratory (p<0.05 for all analysis, Figure 1A, Figure S2A). The mean intralaboratory CV was 14.4% for Aβ1-42, 8.5% for t-tau, and 12.6% for p-tau. For CSF Aβ1-42, the correlation (0.85) and ICC (0.76) were moderate and lower than that of CSF t-tau (*r* = 0.98 and ICC = 0.97) and p-tau (*r* = 0.95 and ICC = 0.90; Table 1, Figure 2A). Internal quality control samples that were analyzed at each measurement also showed higher Aβ1-42 levels, slightly higher t-tau levels, and lower p-tau levels in the second measurement compared to the first measurement (Figure 3). For the clinical analyses 2 lots were used (lot 1 and lot 2), although the majority was measured with the same lot (lot 1). The reanalysis of all samples was performed with another lot (lot 3). Head-to-head comparison of lot 1 and lot 3 for the samples in our study showed that particularly the difference in Aβ1-42 levels between measurements could be explained by lot-to-lot variation although the mean t-tau levels also differed between lot 1 and 3 (Figure 4).

**Table 1:** Agreement between CSF analyses.

	Intralaboratory analyses		Interlaboratory analyses	
	Correlation*	ICC*	Correlation*	ICC*
CSF Aβ1-42	0.85 (0.74–0.91)	0.76 (0.43–0.89)	0.94 (0.91–0.96)	0.92 (0.85–0.96)
CSF t-tau	0.98 (0.97–0.99)	0.97 (0.92–0.99)	0.98 (0.96–0.99)	0.98 (0.96–0.99)
CSF p-tau	0.95 (0.92–0.97)	0.90 (0.53–0.97)	0.94 (0.90–0.96)	0.73 (0.07–0.92)

Results are Pearson correlation and ICC (95% CI) for intra- and interlaboratory CSF analyses. ICC = Intraclass coefficients, ratio = Aβ1-42/t-tau, CSF = cerebrospinal fluid, Aβ = amyloid beta, t-tau = total tau, p-tau = phosphorylated tau.

*All p<0.001.

### Interlaboratory Variability

CSF Aβ1-42 levels were lower, t-tau levels higher, and p-tau levels lower after reanalysis in the second laboratory (p<0.05 for all analysis, Figure 1B, Figure S2B). The interlaboratory CV was 7.3% for Aβ1-42, 6.7% for t-tau, and 27.6% for p-tau. For CSF p-tau, the correlation (0.94) and the ICC (0.73) were high to moderate and lower than that of CSF Aβ1-42 (*r* = 0.94 and ICC = 0.92) and t-tau (*r* = 0.98 and ICC = 0.98; Table 1, Figure 2B).

### Change in AD Classification

**Figure 5:**
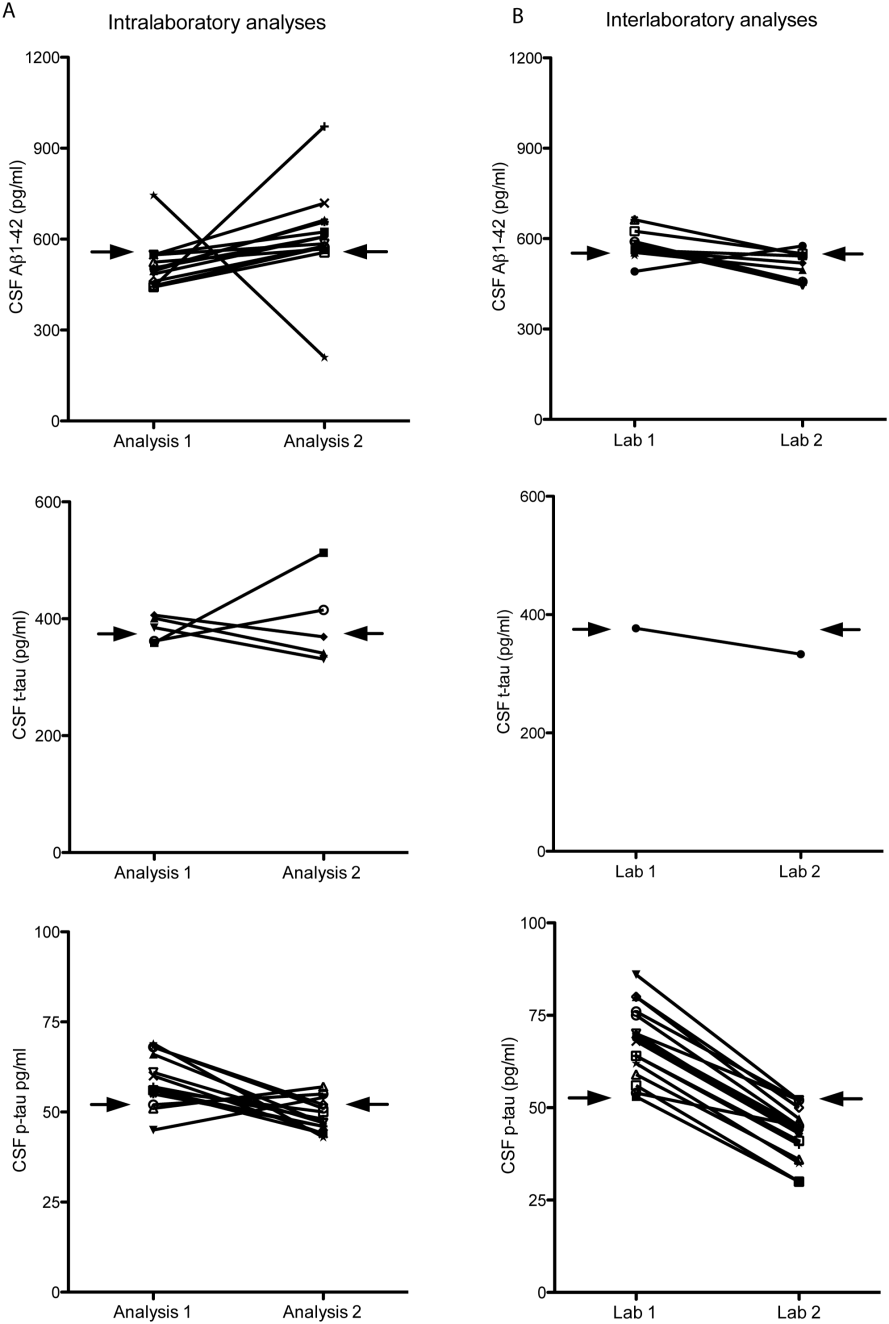
Change in CSF marker classification according to cut-offs. Results are CSF levels only for subjects in which reanalysis let to a different biomarker classification using the cut-offs from Amsterdam to define abnormal CSF values. On the left (A) are changes in biomarker levels for intralaboratory reanalysis and on the right (B) for interlaboratory reanalysis. The arrow represents the applied Amsterdam CSF cut-off. Analysis 1 is routine practice and analysis 2 is performed as part of the LeARN study. CSF = cerebrospinal fluid, Aβ = amyloid beta, t-tau = total tau, p-tau = phosphorylated tau.

We investigated how reanalysis changed the CSF AD classification based on individual CSF markers and based on the combination of CSF markers with an AD profile being defined as abnormal Aβ1-42 and abnormal t-tau or p-tau. Using predefined cutoffs, repeated CSF analyses in the same laboratory led to a change in biomarker classification (normal vs. abnormal) of 26% of subjects based on Aβ1-42, 10% based on t-tau, 29% based on p-tau, and 16% based on the AD profile (Table 2). Repeated CSF analyses in different laboratories using the cut-offs from the Amsterdam lab led to a change in biomarker classification of 12% of subjects based on CSF Aβ1-42, 1% based on t-tau, 22% based on p-tau, and 14% based on the AD profile (Table 2). When we applied lab-specific cut-offs to define an abnormal score, the repeated CSF analyses in different laboratories led to a change in biomarker classification of 17% of subjects based on CSF Aβ1-42, 1% based on t-tau, 12% based on p-tau, and 12% based on the AD profile (Table 2). Figure 5 shows the change in CSF levels for each biomarker for intra- and interlaboratory analyses of subjects in whom reanalysis led to a different biomarker classification as normal vs. abnormal when Amsterdam cut-offs were applied. While most of the subjects with a change in AD classification after reanalysis in the same laboratory as well as in a different laboratory had CSF biomarker values relatively close to the cut-off points, some showed larger changes in CSF biomarker values. The mean change in CSF values for intralaboratory analyses was 177 pg/ml (95% CI 78–275) for Aβ1-42, 72 (13–130) for t-tau, and 12 (9–16) for p-tau. For interlaboratory analyses with the Amsterdam cut-offs, the mean change in CSF biomarker values was 153 pg/ml (57–250) for Aβ1-42 and 26 (20–32) for p-tau. For t-tau, only one subjects showed a change in biochemical diagnosis (a change of 44 pg/ml).

**Table 2:** Numbers of subjects with normal and abnormal classified CSF marker values based on predefined cut-offs.

	Intralaboratory analyses					Interlaboratory analyses								
	Amsterdam cut-offs					Amsterdam cut-offs*					Lab-specific cut-offs**			
Classification of biomarker(s)	Aβ1-42	T-tau	P-tau	AD profile		Aβ1-42	T-tau	P-tau	AD profile		Aβ1-42	T-tau	P-tau	AD profile
Analysis 1 & 2 same classification	74% (37)	90% (45)	71% (35)	84% (42)		88% (67)	99% (75)	78% (59)	86% (65)		83% (63)	99% (75)	88% (67)	88% (67)
Analysis 1 normal & 2 abnormal	2% (1)	4% (2)	6% (3)	-		11% (8)	-	-	9% (7)		16% (12)	-	11% (8)	12% (9)
Analysis 1 abnormal & 2 normal	24% (12)	6% (3)	23% (11)	16% (8)		1% (1)	1% (1)	22% (17)	5% (4)		1% (1)	1% (1)	1% (1)	-

Results are the percentage (number) of subjects with normal and abnormal classified CSF markers based on CSF analysis 1 (routine practice) and analysis 2 (LeARN study). The CSF AD profile was defined as abnormal Aβ1-42 and abnormal t-tau or p-tau, based on predefined cut-offs (see methods).

CSF = cerebrospinal fluid, Aβ = amyloid beta, t-tau = total tau, p-tau = phosphorylated tau.

*The routine analyses in Nijmegen and the LeARN analyses were dichotomized according to the Amsterdam cut-offs (see methods).

**The routine analyses in Nijmegen were dichotomized according to the Nijmegen cut-offs and the LeARN analyses according to the Amsterdam cut-offs (see methods).

## Discussion

We observed clear variability in CSF AD biomarker levels between repeated analyses in the same laboratory as well as between two different laboratories. Our study is the first to show that this variability frequently led to a change in CSF-based AD diagnosis when predefined cut-offs for abnormal CSF values were applied.

CSF intralaboratory variability (based on CV) was largest for Aβ1-42, consistent with previous studies [5]–[7]. Change in AD-like scores after repeated analyses in the same laboratory was, however, highest for CSF p-tau (29%) followed by Aβ1-42 (26%) and t-tau (10%). This large change for p-tau is likely due to a smaller range of values of p-tau and values being closer to the cut-off compared to other markers, such that a small change in observed concentration more easily leads to a different classification.

CSF interlaboratory variability (based on CV) was largest for p-tau, unlike findings from previous studies, which found that interlaboratory variability was largest for Aβ1-42 [5]–[7]. Change in AD-like scores after repeated analyses in different laboratories was also higher for CSF p-tau (22%) than for Aβ1-42 (12%) and t-tau (1%) when the same cut-offs were used for each site. A rather unexpected finding was that the interlaboratory variability of Aβ1-42 was smaller than the intralaboratory variability.

A major finding was that CSF analytical variability frequently led to a change in diagnostic CSF-based AD classification. Importantly, also a diagnosis of a CSF AD profile based on Aβ1-42 and t-tau or p-tau changed in 12–16% of the cases. As some of the subjects whose AD classification changed after reanalysis had values around the cut-off points, it could be helpful to use a range around a cut-off point rather than a fixed cut-off point.

The intra- and interlaboratory variability in CSF results can result from differences in preanalytical and analytical procedures, and lot-to-lot variation of analytical kits [9], [15]. The intralaboratory variability is likely mainly due to lot-to-lot variation as other procedures remained essentially the same. Lot-to-lot variation is a technical limitation of the ELISA method, which should also be considered when defining cut-offs and interpreting CSF values close to the cut-off. Additional support for this comes from our observation that internal control values showed a striking lot-to-lot variation of up to 20%. The change in internal control values was of the same order of magnitude and same direction of change as the change in CSF scores from the patient samples. Head-to head comparison of lots for the samples in our study showed that mainly CSF Aβ1-42 variability could be explained by lot-to-lot variation. This likely explains why CSF intralaboratory variability was largest for Aβ1-42. Another possible explanation for the intralaboratory variability is variability in freezing conditions and storage time. However, previous studies showed that these factors have a minor impact on variability of CSF values [16], [17].

Interlaboratory variability may also be caused by lot-to-lot variation. Indeed, lots used in each lab showed only minor overlap (data not shown). Differences in analytical procedures may also have contributed to the interlaboratory variability. However, both laboratories used similar protocols, were both trained in a hands-on workshop [9], and were similarly experienced.

Another source of variability between laboratories, which may influence CSF-based AD classification, is the difference in cut-offs used. We, therefore, tested interlaboratory variability for CSF-based AD classification both with the same CSF cut-offs as well as lab-specific cut-offs. While lab-specific cut-offs for Aβ1-42 and t-tau did not differ much, there was a large difference for p-tau (85 pg/ml in Nijmegen vs. 52 pg/ml in Amsterdam). This was also reflected in the interlaboratory variability in CSF-based AD classification. Using the same cut-off, abnormal p-tau was more common when samples were analyzed in Nijmegen than in Amsterdam. Change in classification was often due to subjects with scores around the cut-off (Figure 5B). Using the lab-specific cut-off, however, abnormal p-tau was more common when samples were analyzed in Amsterdam than in Nijmegen. Here change in classification was mainly due to differences in cut-offs. This clearly indicates that lab-specific cut-offs may also influence comparability between laboratories.

Our study has several limitations. The cut-offs that were used may have influenced our findings on change in CSF-based AD diagnosis. However, as no universal CSF cut-offs are available, we applied routinely used validated cut-offs.

One of the major strengths of this study was the large number of CSF samples used to study interlaboratory variability, as most previous studies were based only on a few samples [5]–[7]. Furthermore, our study is the first to show head-to-head comparison of lots within one center to directly address the issue of lot-to-lot variability. Therefore, our findings may provide a valuable addition to the described findings of the Alzheimer Association Quality Control program. In addition, our study design allows generalization to other CSF centers that analyze CSF AD biomarkers using ELISA, as it reflects CSF procedures in general clinical practice.

Together, our findings suggest that variability in CSF analyses is common between and within laboratories, in particular for Aβ1-42 and p-tau. A substantial part of this variability seems to be explained by lot-to-lot variation of analytical kits. The variability has a large impact on CSF-based AD diagnosis or treatment decisions in clinical settings, suggesting that we should be careful when interpreting CSF findings and always interpret them within a clinical context [18], and with reference to internal standards. Also the use of age-adjusted cut-offs may be helpful, as tau levels are known to increase with age.

For the moment this rightly restricts the indication for CSF biomarker testing in diagnostic guidelines as complimentary and non-obligatory [19], [20]. The recent consensus on standardization of preanalytical aspects of CSF analyses [15], [17], as well as the ongoing worldwide quality control study [6], and standardization projects (www.neurodegenerationresearch.eu) will help to move towards a standardized and harmonized implementation of CSF markers in clinical routine.

## Supporting Information

Figure S1 **Laboratories that performed CSF analyses for clinical routine are presented on the left (analysis 1); the laboratory that performed CSF analyses for the LeARN study is presented on the right (analysis 2).**
(DOCX)

Figure S2 **Change in CSF levels after reanalysis.** Results on the left (A) are changes in CSF biomarker levels for intralaboratory reanalysis and on the right (B) for interlaboratory reanalysis. Analysis 1 is routine practice and analysis 2 is performed as part of the LeARN study. CSF = cerebrospinal fluid, Aβ = amyloid beta, t-tau = total tau, p-tau = phosphorylated tau.
(DOCX)

Table S1 **Baseline patient demographics.** Results are mean (SD) or number (%), presented for the total sample and separately for the sample for intralaboratory analyses (Amsterdam cohort) and the sample for interlaboratory analyses (Maastricht & Nijmegen cohort). MMSE = Mini-Mental State Examination, CDR = Clinical dementia rating scale, SCI = subjective cognitive impairment, MCI = mild cognitive impairment, AD = Alzheimer’s disease.
(DOCX)
