# Supplementary material for: Variability of CSF Alzheimer’s Disease Biomarkers: Implications for Clinical Practice
Source: PLoS One. 2014 Jun 24;9(6):e100784. doi: 10.1371/journal.pone.0100784 (PMC4069102; doi:10.1371/journal.pone.0100784)
Supplement: Figure S1 — Laboratories that performed CSF analyses for clinical routine are presented on the left (analysis 1); the laboratory that performed CSF analyses for the LeARN study is presented on the right (analysis 2). (DOCX) [file pone.0100784.s001.docx]

Supplemental Figure S1.
