# Supplementary material for: Variability of CSF Alzheimer’s Disease Biomarkers: Implications for Clinical Practice
Source: PLoS One. 2014 Jun 24;9(6):e100784. doi: 10.1371/journal.pone.0100784 (PMC4069102; doi:10.1371/journal.pone.0100784)
Supplement: Figure S2 — Change in CSF levels after reanalysis. Results on the left (A) are changes in CSF biomarker levels for intralaboratory reanalysis and on the right (B) for interlaboratory reanalysis. Analysis 1 is routine practice and analysis 2 is performed as part of the LeARN study. CSF = cerebrospinal fluid, Aβ = amyloid beta, t-tau = total tau, p-tau = phosphorylated tau. (DOCX) [file pone.0100784.s002.docx]

Supplemental Figure S2. Change in CSF levels after reanalysis
