# Supplementary material for: Variability of CSF Alzheimer’s Disease Biomarkers: Implications for Clinical Practice
Source: PLoS One. 2014 Jun 24;9(6):e100784. doi: 10.1371/journal.pone.0100784 (PMC4069102; doi:10.1371/journal.pone.0100784)
Supplement: Table S1 — Baseline patient demographics. Results are mean (SD) or number (%), presented for the total sample and separately for the sample for intralaboratory analyses (Amsterdam cohort) and the sample for interlaboratory analyses (Maastricht & Nijmegen cohort). MMSE = Mini-Mental State Examination, CDR = Clinical dementia rating scale, SCI = subjective cognitive impairment, MCI = mild cognitive impairment, AD = Alzheimer’s disease. (DOCX) [file pone.0100784.s003.docx]

|  | Total sample (n=126) | Amsterdam cohort (n=50) | Maastricht & Nijmegen cohort (n=76) |
| --- | --- | --- | --- |
| Age | 67.0 (9.2) | 65.0 (7.7) | 68.3 (9.8) |
| Female, n | 36 (29%) | 12 (24%) | 24 (32%) |
| MMSE | 26.1 (2.9) | 25.2 (3.0) | 26.8 (2.7) |
| CDR | 0.6 (0.3) | 0.7 (0.4) | 0.5 (0.3) |
| Clinical diagnosis, n |  |  |  |
| SCI | 41 (33%) | 9 (18%) | 32 (42%) |
| MCI | 33 (26%) | 8 (16%) | 25 (33%) |
| AD-type dementia | 32 (25%) | 17 (34%) | 15 (20%) |
| Other dementia | 20 (16%) | 16 (32%) | 4 (5%) |

Supplemental Table S1. Baseline patient demographics

Results are mean (SD) or number (%), presented for the total sample and separately for the sample for intralaboratory analyses (Amsterdam cohort) and the sample for interlaboratory analyses (Maastricht & Nijmegen cohort). MMSE=Mini-Mental State Examination, CDR=Clinical dementia rating scale, SCI=subjective cognitive impairment, MCI=mild cognitive impairment, AD=Alzheimer’s disease.
